# Antiemetic Strategies in Patients Who Undergo Hematopoietic Stem Cell Transplantation

**DOI:** 10.1007/s44228-022-00012-8

**Published:** 2022-07-11

**Authors:** Sayako Yuda, Shigeo Fuji, Bipin Savani, Katie S. Gatwood

**Affiliations:** 1 Department of Hematology, Osaka International Cancer Institute, 3-1-69, Otemae, Chuo-ku, Osaka-city, Osaka 5418567 Japan; 2 Stem Cell Transplant and Cellular Therapy, Vanderbilt University Medical Center, Nashville, USA

**Keywords:** Antiemetics, Hematopoietic cell transplantation

## Abstract

Hematopoietic stem cell transplantation (HSCT) is an integral part of the treatment strategy in patients with a hematological disorder. Chemotherapy-induced nausea and vomiting (CINV) is still an issue in patients who undergo HSCT. While several guidelines for the antiemetic therapy against CINV have been published, there is no detailed information about appropriate antiemetic drugs for each conditioning regimen in HSCT. Various studies reported that the triplet of 5-HT3RA, NK1RA, and dexamethasone appears useful in HSCT. However, each antiemetic has unique adverse effects or interactions with specific drugs. Here, we review the literature relating to clinical trials on the prevention of CINV, and summarize the information to clarify the benefit of antiemetic regimens.

## Introduction

Nausea and vomiting are common side effects of chemotherapeutic drugs [1, 2]. There is no doubt that high-dose chemotherapies used as conditioning regimens in hematopoietic stem-cell transplantation (HSCT), especially total body irradiation (TBI) or high-dose cyclophosphamide, have high emetic potential [3–5]. The incidence of chemotherapy-induced nausea and vomiting (CINV) has dramatically decreased with the advent of a variety of antiemetics, such as the new generation 5-hydroxy tryptamine3 receptor antagonist (5-HT3RA), the neurokinin-1 receptor antagonist (NK1RA) for highly emetic chemotherapy (HEC), and the addition of antipsychotic agents, such as olanzapine. However, CINV is still a concern in HSCT recipients [6], and can be associated with a substantially impaired quality of life (QoL) and a need for artificial feeding to prevent malnutrition.

The first proposal for controlling CINV was published in 1997 [7] and has been continuously revised by a variety of cancer societies, including the American Society of Clinical Oncology (ASCO) [8], the Multinational Association of Supportive Care in Cancer (MASCC), the European Society for Medical Oncology (ESMO) [9] and the National Comprehensive Cancer Network (NCCN) [10]. Although some guidelines describe antiemetic use in the setting of high-dose chemotherapy for HSCT [8, 9, 11], there is no detailed recommendation on their use in each conditioning regimen, and according to the difference in the severity of CINV among the various regimens. This becomes especially challenging to interpret, as several agents used for HSCT conditioning are labeled as being of low or moderate emetic risk, although this classification is typically based on non-HSCT dosing of these agents [12]. Additionally, it does not account for the use of multiple moderately emetic agents in combination which, in many cases, leads the regimen to be considered highly emetic. Therefore, we expect that the clinical practice of antiemetic prescribing for HSCT differs among different countries/institutions, which means that the compliance with the antiemetic guidelines are not necessarily as high in this field as in non-HSCT setting [13–16]. In this article, we review the published literature and summarize the information to clarify the benefit of antiemetic regimens.

## Literature Review

A review of the literature reporting on antiemetics in HSCT was conducted. The PubMed database was searched to identify all the articles relating to antiemetics in HSCT.

## Impact of CINV Control in HSCT on Nutritional Status

**Fig. 1:**
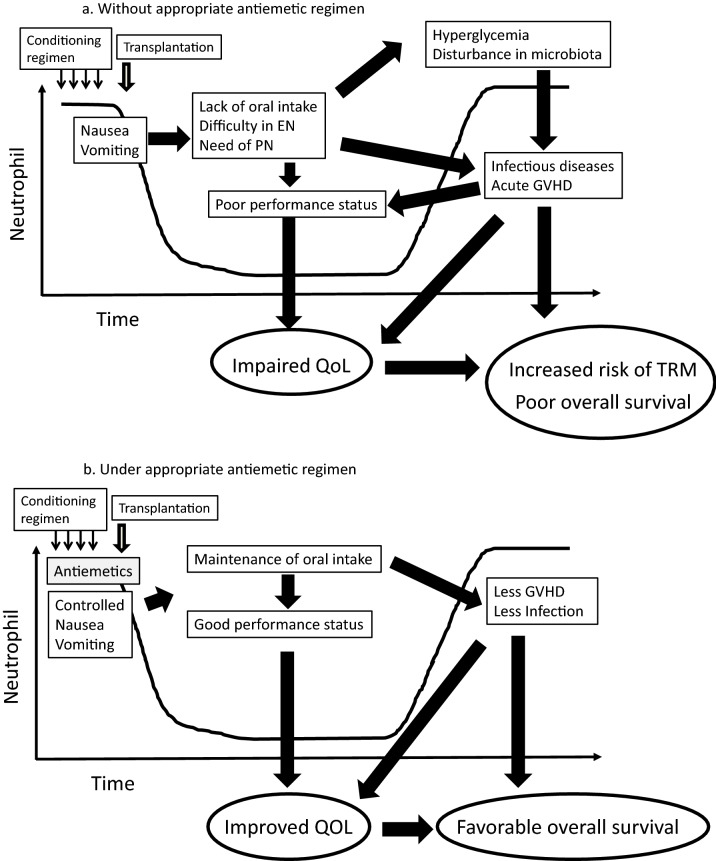
Impact of antiemetics on the clinical outcome after HSCT **a** without appropriate antiemetic regimen, **b** under appropriate antiemetic regimen. *EN* enteral nutrition, *PN* parenteral nutrition, *GVHD* graft-versus-host disease, *QoL* quality of life, *TRM* transplant-related mortality

CINV control is important for patients who receive any form of chemotherapy, particularly from the viewpoint of maintenance of QoL and prevention of malnutrition after HSCT (Fig. 1a and b) [17–19]. Recent reports which assessed the relationship between patients’ general condition and treatment-related complications in HSCT implied that antiemetic treatment has a significant influence on the clinical course of patients receiving HSCT [20, 21].

One important aim of antiemetics is the maintenance of oral caloric intake after HSCT. Nutritional support during the early phase after HSCT is crucial to maintaining body weight and performance status [21–23]. Artificial feeding, such as enteral nutrition (EN) or parenteral nutrition (PN), is used to maintain the target caloric intake in patients with insufficient oral intake due to nausea or vomiting after HSCT. The presence of nausea or vomiting hampers oral intake, as well as the application of EN after HSCT [22]. Feeding through the gastrointestinal (GI) tract is considered important to facilitate the recovery from GI tract damage and, possibly, to maintain the microbiota status after HSCT [24, 25]. Several studies showed that a balanced intestinal flora affects the human immune system and reduces the risk of graft-versus-host disease (GVHD) [26–28]. Retrospective studies suggested the beneficial impact of EN after HSCT [29–33]. In addition, the use of PN is associated with side effects such as hyperglycemia, infection, and liver dysfunction. As hyperglycemia is reported to be associated with increased gut permeability, poor wound healing, and impaired neutrophil function [34–36], excessive use of PN should be avoided. Though no study directly assessed whether CINV affects the incidence of transplant-related complications and death after HSCT, it is reasonable to provide sufficient antiemetics to prevent nausea and vomiting in patients with HSCT, considering the benefit to maintain adequate oral intake after HSCT.

## Previous Studies on Antiemetics in HSCT

According to the major guidelines, recommendations on antiemetics in patients who received high-dose chemotherapy before HSCT are limited (Table 1). There is no description of the difference or adjustment according to the type or dose of drugs used for HSCT conditioning regimens. Thus, it is unclear whether we should use the same antiemetics in a classical myeloablative conditioning regimen like cyclophosphamide plus TBI, in a modern myeloablative but reduced-toxicity regimen such as fludarabine plus busulfan or melphalan, and in a reduced-intensity conditioning regimen [8, 9, 11]. It is expected that different antiemetic regimens for each conditioning regimen should be used in clinical practice. While notable developments in HEC management in other fields are addressed in current updates [8, 9, 11], antiemetic strategy in HSCT has not yet been well established. Thus, here we review research featuring the use of antiemetic therapy in HSCT.

**Table 1:** Summary of the recommendation on prophylactic antiemetics in guidelines

Therapy	Antiemetics combination
High-dose chemotherapy in HSCT	NK-1 receptor antagonist
	5-HT3 receptor antagonist
	Dexamethasone
	Olanzapine (optional)
HEC	NK-1 receptor antagonist
	5-HT3 receptor antagonist
	Dexamethasone
	Olanzapine
MEC exculding regimens with carboplatin AUC > 4 mg/mL/minute	5-HT3 receptor antagonist
	Dexamethasone
Low-emetic risk chemotherapy	5-HT3 receptor antagonist or dexamethasone
Minimal-emetic risk chemotherapy	not necessary

*HSCT* hematopoietic stem cell transplantation, *NK-1* neurokinin-1, *5-HT3* 5-hydroxy tryptamine3, *HEC* highly emetic chemotherapy, *MEC* highly emetic chemotherapy, *AUC* area under the curve

## Emetic Risk of Each Drug Used for Conditioning Regimens in HSCT

Unique chemotherapeutic drugs are used in conditioning regimens for HSCT. The drugs may be used in higher doses or in combination in the setting of HSCT. These factors make it difficult to define the emetic risk of certain drugs used for conditioning regimens. For instance, there are inconsistent recommendations in the guidelines for chemotherapy with busulfan or melphalan. First, there is no recommendation regarding intravenous melphalan in the ASCO/MASCC/ESMO guidelines, but in the NCCN guideline it is classified as of moderate risk [1, 37]. Second, intravenous busulfan is treated as of moderate risk in the ASCO and NCCN guidelines [1], but minimal in the MASCC/ESMO guidelines [37]. Previously, intravenous busulfan was treated as minimal risk in the ASCO guideline. These inconsistencies could be confusing for clinicians who are responsible for the determination of antiemetics regimens for given conditioning regimen protocols.

## Each antiemetic drug in HSCT

### Prevention of Acute and Delayed Emesis

#### 5-HT3RA

Since the demonstration that granisetron was effective against CINV in HEC in the non-HSCT setting decades ago [38], many researchers conducted clinical trials on administration of 5-HT3RAs in HSCT. As many reports revealed the efficacy and safety of 5-HT3RAs in classical myeloablative conditioning regimens using TBI, 5-HT3Ras are widely applied in the HSCT field [39]. In terms of the type of 5-HT3RA, the superiority of palonosetron in comparison to first-generation 5-HT3RA is still controversial [40], but it is obvious that 5-HT3RAs are safe enough to be used in the HSCT setting [40–42]. Now, 5-HT3RAs have been regarded as a basic part of antiemetic strategy for all HSCT conditioning regimens.

#### Dexamethasone

Dexamethasone is used in almost all cases as a standard component of antiemetics for high-dose chemotherapy for HSCT [1, 39], although the exact mechanism of action of corticosteroids for CINV prevention is unclear.

Though dexamethasone plays a vital role in CINV treatment, the appropriate dose and schedules for dexamethasone remain unidentified. When a higher dose of dexamethasone is used, it can be associated with an increased risk of side effects such as hypertension, glucose intolerance, and others [39]. Challenges to dose-reduction of dexamethasone in HEC or moderate-emetic chemotherapy (MEC) have been reported. Some of the clinical trials on MEC or HEC without cisplatin suggested that the administration of palonosetron, instead of the first-generation 5-HT3RAs, made it possible to decrease the dose of dexamethasone to 8 mg on day 1 and to omit it on subsequent days [43, 44]. Additionally, the NCCN guideline on antiemesis updated in 2018 mentioned that dexamethasone on days 2–4 in HEC can be replaced with olanzapine. A further complicating factor of the use of dexamethasone in allogeneic HSCT conditioning regimens is its potential immunomodulatory effects. Excessive steroid use in allogeneic transplantation can lead to compromised engraftment and increased infection risk [45]. Particularly in the setting of haploidentical transplantation with post-transplant cyclophosphamide, the administration of steroids as antiemetics from the day of HSCT until the administration of cyclophosphamide may compromise the efficacy of post-transplant cyclophosphamide, lead to increased risk of GVHD and, therefore, is generally recommended to be avoided as routine CINV prophylaxis [46].

### NK1RA

In the 2000s, NK1RA aprepitant was first introduced as an effective drug against CINV in HEC. It was reported that aprepitant remarkably reduced the incidence of CINV [8, 9, 11]. Many studies on antiemetic strategy in HSCT revealed efficacy and safety of NK-1 inhibitors in a combination of 5-HT3RA and dexamethasone (“triplet”) during the conditioning chemotherapy [47–59]. A randomized placebo-controlled trial on CINV during high-dose chemotherapy in autologous (auto-) or allogeneic (allo-) HSCT reported that complete response rates, defined as no emesis with no or mild nausea, were 81.9% in triplet arm and 65.8% in doublet arm (*P* < 0.001) [56]. Percentages of patients with no emesis were 73.3% for aprepitant and 22.5% placebo (*P* < 0.001). Schmitt et al. proved the triplet to be effective in preventing CINV caused by high-dose melphalan followed by auto-ASCT administered to patients with multiple myeloma [58]. Complete response rates, defined as no emesis and no rescue therapy within 120 h of melphalan administration, were 58% in the aprepitant and 41% in the placebo arm (odds ratio 1.92 [1.23, 3.00], *P* = 0.0042). It was confirmed that the addition of aprepitant was tolerable and did not change the pharmacokinetics and metabolism of calcineurin inhibitors or antineoplastic agents [60–62]. Of note, aprepitant is a moderate inhibitor of CYP3A4 and therefore has drug interaction potential with both chemotherapy and supportive care agents used in HSCT [63]. In particular, based on available evidence, it is recommended to use aprepitant with caution or to avoid regimens containing busulfan and etoposide, as they are metabolized via CYP3A4 [64].

Rolapitant and netupitant are other NK1RA. Phase III trials with these agents showed promising data in HEC [65–67], and the NCCN guideline [11] recommended their use in MEC as well as HEC. However, clinical data on these two NK1RAs in HSCT have not been published.

#### Olanzapine

While central dopamine receptor antagonists, including antipsychotics, are effective for CINV, they usually cause not only somnolence but also extrapyramidal disorders, such as tremor and akathisia, which reduce the QOL and ADL of patients. The incidence of extrapyramidal disorder has been decreased by using new types of antipsychotics including serotonin-dopamine antagonists (SDA) and multi-acting receptor-targeted antipsychotics (MARTA). As olanzapine, an agent classified as MARTA, is potent in controlling CINV [18, 68, 69], the latest guidelines recommend it for the patients with HEC in non-HSCT setting [8, 9, 11]. It is also important to consider that olanzapine causes QTc prolongation and this can have an additive effect with many other therapies used routinely in HSCT.

In recent years, some reports on CINV in HSCT focusing on olanzapine were published [70–73]. The FOND-O trial investigated whether the addition of olanzapine 10 mg on each chemotherapy day and 3 days after the triplet prevents CINV in patients with hematological malignancies under HEC or conditioning therapy for HSCT [73]. While there was no significant difference in terms of prevention of CINV in the early phase, the complete response rate was higher in patients who received olanzapine than in those who did not: 55% versus 26% in the overall assessment period and 60.8% versus 30% in the delayed phase. However, subgroup analysis showed that the addition of olanzapine significantly improved CINV control in the auto-HSCT but not in the allo-HSCT cohort, possibly due to a limited number of cases in the subgroup analysis, which should be determined in larger studies in the future. Nakagaki et al. reported that olanzapine was effective to treat breakthrough CINV [72]. Sixty-two patients enrolled in that study receiving auto- or allo-HSCT following high-dose chemotherapy were administered the standard triplet prophylaxis, with added either first or second-generation 5-HT3 antagonists or olanzapine, as rescue-medication against breakthrough emesis. Both trials concluded that olanzapine was tolerable for HSCT patients and did not affect the engraftment of hematopoietic stem cells.

In summary, standard prophylaxis for CINV in HSCT appears to be the triplet of a 5-HT3RA, an NK1RA, and dexamethasone, even though it is less effective for patients receiving high-dose chemotherapy in HSCT than those with HEC or MEC [74]. Even in HSCT with reduced-intensive conditioning (RIC) regimens, which are usually regarded as having a lower risk for nausea, it is unclear whether we should use antiemetics for HEC or MEC. As only few studies have evaluated antiemetic treatment focusing on a specific conditioning regimen in HSCT [41, 47, 48, 54, 58, 59, 61, 70, 75], more evidence to develop a better antiemetic strategy for each conditioning regimen is needed.

## Treatment for Breakthrough Emesis

In the absence of specific data, the management of breakthrough emesis in HSCT is similar to that of standard chemotherapy. However, physicians must note that prolonged nausea and vomiting in HSCT are often caused not only by conditioning regimens but also other causes like infection, GVHD, primary disease in the GI tract, or concomitantly used drugs.

In cases with a limited improvement of CINV by pharmacological approaches, some research suggested that refractory emesis was managed by using complementary medicine (CAM), including acupuncture [76] and aromatherapy [77]*.* It is unclear whether CAM in immune-compromised patients could be safely applied, which should be determined in the setting of HSCT.

## How to Choose the Antiemetics in HSCT

As mentioned above, the current guidelines for antiemetics in HSCT do not dictate the detailed management recommendations for each conditioning regimen, As summarized in Table 2, doses and schedules of conditioning regimens are much more complicated than those of HEC in the non-HSCT setting. Thus, it is practically difficult to give the ranking of CINV risk to the overall regimen. It must be essential to make sophisticated plans for antiemetics, which are adapted to the respective conditioning regimens.

**Table 2:** Emetic risk of conditioning regimens according to guidelines

Regimens	Dose	Risk category
		Category as a single agent
Standard regimens*		
Cy/TBI		
Cy	60 mg/kg/day × 2 days	High
TBI	2 Gy × 2/day × 3 days	High
Bu/Cy		
Bu	3.2 mg/kg/day × 4 days	Moderate
Cy	60 mg/kg/day × 2 days	High
BEAM		
BCNU	300 mg/m^2^/day × 1 day	High
VP	200 mg/m^2^/day/4 days	Low
AraC	200 mg/m^2^ × 2/day/4 days	Low
MEL	140 mg/m^2^/day × 1 day	Moderate
MEAM		
MCNU	300 mg/m^2^/day × 1 day	High
VP	200 mg/m^2^/day/4 days	Low
AraC	200 mg/m^2^ × 2/day/4 days	Low
MEL	140 mg/m^2^/day × 1 day	Moderate
LEED		
MEL	130 mg/m^2^/day × 1 day	Moderate
VP	300 mg/m^2^/day × 3 days	Low
Cy	60 mg/kg/day × 2 days	High
Dexa	40 mg/day × 4 days	–
MEL		
MEL	100 mg/m^2^/day × 2 days	Moderate
Bu/MEL		
Bu	3.2 mg/kg/day × 4 days	Moderate
MEL	140 mg/m^2^/day × 1 day	Moderate
Intensified regimens*		
Cy/TBI/VP		
Cy	60 mg/kg/day × 2 days	High
TBI	2 Gy × 2/day × 3 days	High
VP	30–60 mg/m^2^/day × 1 day	Low
Cy/TBI/AraC		
Cy	60 mg/kg/day × 2 days	High
TBI	2 Gy × 2/day × 3 days	High
AraC	2–3 g/m^2^ × 2/day × 2 days	Moderate
Cy/TBI/TT		
Cy	60 mg/kg/day × 2 days	High
TBI	2 Gy × 2/day × 3 days	High
TT	5 mg/kg/day × 2 days	Moderate
Bu/Cy/MEL		
Bu	3.2 mg/kg/day × 4 days	Moderate
Cy	60 mg/kg/day × 2 days	High
MEL	140 mg/m^2^/day × 1 day	Moderate
AraC/TBI		
AraC	3 g/m^2^ × 2/day × 2 days	Moderate
TBI	2 Gy × 2/day × 3 days	High
VP/TBI		
VP	60 mg/kg/day × 1 days	High
TBI	2 Gy × 2/day × 3 days	High
Reduced toxicity regimens*		
Flu/MEL		
Flu	25 mg/m^2^/day × 5 days	Minimal
MEL	140 mg/m^2^/day × 1 day	Moderate
Flu/Cy		
Flu	25 mg/m^2^/day × 5 days	Minimal
Cy	60 mg/kg/day × 2 day	High
Flu/Bu4		
Flu	30 mg/m^2^/day × 6 days	Minimal
Bu	3.2 mg/kg/day × 4 days	Moderate
Flu/Bu2		
Flu	30 mg/m^2^/day × 6 days	Minimal
Bu	3.2 mg/kg/day × 2 days	Moderate

*CY* cyclophosphamide, *TBI* total body irradiation, *Bu* busulfan, *BCNU* carmustine, VP etoposide, *AraC* cytarabine, *MEL* melphalan, *MCNU* ranimustine, *Flu* fludarabine

*Listed in the order of emetic risk, highest first

Since the extent of emesis changes day by day, physicians should reconsider the plan for antiemetics every day. Guidelines recommend that patients with multi-day chemotherapy should be offered antiemetics which are appropriate for the risk of the agents administered on each day and for two to three days after the completion of the regimens [8, 9].

NK1 RA, which is effective for both acute and delayed CINV, should be used to target the days with the highest risk for CINV. However, physicians must keep in mind that the information about drug interaction between antiemetics and chemotherapy agents and therapies for supportive care in HSCT is limited as compared with that in HEC.

Though dexamethasone is one of the most convenient antiemetics for clinical use because of less interaction with cytotoxic drugs, extra use of steroids may be intolerable to some patients because of metabolic disorders, such as hyperglycemia and hypoalbuminemia, which overlaps with side effects from the calcineurin inhibitors. Frequent and prolonged exposure to steroid agents causes immunodeficiency, leading patients to severe infection. Steroid administration before post-transplant cyclophosphamide is avoided as it reduces its effects. The control of emesis in post-transplant cyclophosphamide using 5-HT3RA and NK1RA was reported to be insufficient [78]. Individualization of dexamethasone use must be critical, especially in HSCT.

The study which assessed the beneficial impact of olanzapine is still limited [79]. We need more data to assess the safety and efficacy of olanzapine in combination with other antiemetics in HSCT. It is known that olanzapine also causes metabolic disorders. Studies on HEC reported that some of the patients treated with olanzapine developed hyperglycemia [18, 69].

Moreover, it is essential to take into account the CINV risks. Previous studies demonstrated that female gender, young age, no history of alcohol consumption, no smoking habit, prior episodes of pregnancy-related morning or motion sickness, and poor performance status are high-risk factors for CINV [80–82]. At the time of planning for antiemetics in HSCT, physicians/pharmacists should reevaluate any CINV which the patients developed during their prior chemotherapy. It is also important to distinguish CINV from symptoms related to other causes, such as side effects by opioids, gastrointestinal infections, gut GVHD, and central nervous system infiltration by malignancy.

## Conclusion

In summary, the adequate dose and schedule of antiemetics against CINV in HSCT have not been established. Antiemetic strategy in HSCT should be individualized taking into consideration the patients’ characteristics and risk categories of each regimen.
